# Exploration of Freshness Identification Method for Refrigerated Vegetables Based on Metabolomics

**DOI:** 10.3390/metabo14120665

**Published:** 2024-12-01

**Authors:** Zixuan Meng, Haichao Zhang, Jing Wang, Lianfeng Ai, Weijun Kang

**Affiliations:** 1 School of Public Health, Hebei Medical University, Shijiazhuang 050017, China; 22033100218@stu.hebmu.edu.cn; 2 Shijiazhuang Customs Technology Center, Shijiazhuang 050051, China; haichao0602@163.com (H.Z.); wangjingid@126.com (J.W.); 3 Hebei Key Laboratory of Environment and Human Health, Shijiazhuang 050017, China

**Keywords:** metabolomics, vegetables, refrigeration transportation, amino acid quantitative detection

## Abstract

**Background**: The rapid development of refrigerated transportation technology for fresh vegetables has extended their shelf life. Some vegetables may appear undamaged on the surface, but their freshness may have decreased, often resulting in the phenomenon of passing off inferior vegetables as good. It is very important to establish a detection method for identifying and assessing the freshness of vegetables. **Methods**: Therefore, based on metabolomics methods, this study innovatively employed UHPLC-Q-Exactive Orbitrap MS and GC–MS techniques to investigate the metabolites in the refrigerated storage of four vegetables, namely chard (*Beta vulgaris* var. cicla L), lettuce (*Lactuca sativa* var. ramose Hort.), crown daisy (*Glebionis coronaria* (L.) Cass. ex Spach), and tomato (*Solanum lycopersicum* L.), exploring key biomarkers for assessing their freshness. UPLC-TQ MS was used for the quantitative analysis of key metabolites. **Results**: The results showed that arginine biosynthesis and the metabolism of alanine, aspartate, and glutamate are key pathways in vegetable metabolism. Four key metabolites were selected from chard, five from lettuce, three from crown daisy, and five from tomato. **Conclusions**: Comparing the content of substances such as alanine and arginine can help infer the freshness and nutritional value of the vegetables, providing important references for detecting spoilage, determining storage time, and improving transportation conditions. This research holds significant relevance for the vegetable transportation industry.

## 1. Introduction

China is the world’s largest producer and consumer of vegetables, offering its citizens a wide range of food choices. As living standards improve, consumers are increasingly concerned about the quality and safety of vegetables. The market demand for fresh and nutritious vegetables is growing stronger, placing higher demands on storage, transportation, and preservation technologies. This is particularly true for vegetables like lettuce and tomatoes, which are often consumed fresh and raw and whose preservation techniques still face many challenges [1,2,3]. Currently, the vegetable storage and transportation industry is vigorously developing refrigerated transportation methods to meet the national demand for fresh and safe vegetables. The cold chain circulation process of fresh vegetables is stored at a temperature of around 0 degrees Celsius. Under low-temperature conditions, the respiration rate of vegetables is reduced, metabolism slows down, and shelf life is extended [4,5]. However, the metabolic processes continue, leading to changes in active substances and nutrient loss. Some vegetables may appear undamaged on the surface, but their freshness can significantly decline, resulting in issues like the passing off of inferior products as high quality. Establishing a detection method to identify and assess the freshness of vegetables is crucial to ensuring consumers receive fresh and nutritious food.

In recent years, omics-based plant research techniques have made great strides, and metabolomics has emerged as a new method for exploring patterns of metabolite changes [6,7]. Non-targeted metabolomics employs high-throughput techniques to analyze a wide range of metabolites, providing qualitative and relative quantitative evaluations of chemical components in complex extracts and allowing for the study of correlations between metabolites [8,9]. Targeted metabolomics can accurately quantify identified results to validate experimental findings and further speculate on metabolic processes. The combination of these approaches can help explore the reasons behind changes in vegetable taste, study mechanisms of deterioration, assess changes in metabolite content, and identify different varieties [10,11]. Currently, technologies such as liquid chromatography–tandem mass spectrometry (LC–MS/MS) and gas chromatography–tandem mass spectrometry (GC–MS) effectively analyze and identify multiple metabolites. For example, Liu et al. [12] used a non-targeted metabolomics method based on liquid chromatography-mass spectrometry (LC–MS) to study the browning mechanism of fresh-cut eggplants, discovering that the content of 946 metabolites changed dynamically over time, with metabolites showing similar trends and sharing common metabolic pathways. Guo et al. [13] identified thousands of metabolites in tomatoes using UHPLC-QTOF-MS technology, finding that more total sugars, acids, and flavonoids accumulated during storage. However, relying on a single technique for analyzing metabolite types and trends often cannot provide comprehensive research information. GC–MS is suitable for analyzing low-polarity, low-boiling-point, and derivatized low-boiling-point compounds, while LC–MS is the main method for separating secondary metabolites from plants. By combining these two technologies, researchers can obtain more comprehensive material information and conduct detailed differential analyses for optimal results [14,15].

This study is based on metabolomics technology, employing ultra-high-performance liquid chromatography quadrupole electrostatic field orbital ion trap mass spectrometry (UHPLC-Q-Orbitrap-MS) and gas chromatography–mass spectrometry (GC–MS) in conjunction with statistical analysis to investigate the freshness of vegetables after refrigeration. LC–MS/MS was used for the quantitative analysis of key differences. Vegetables can be classified in various ways. According to the different edible parts, vegetables can be divided into root vegetables, stem vegetables, leafy vegetables, fruit vegetables, etc. According to the method of consumption, they can also be classified into raw and cooked vegetables. To ensure the representativeness of the samples, four types of vegetables were selected as research subjects: leafy vegetables such as chard (*Beta vulgaris* var. cicla L.) and lettuce (*Lactuca sativa* var. ramose Hort.), stem vegetables such as crown daisy (*Glebionis coronaria* (L.) Cass. ex Spach), and fruit vegetables such as tomatoes (*Solanum lycopersicum* L.). Lettuce and tomato were chosen as representatives of raw edible vegetables, while crown daisy and chard were chosen as representatives of cooked vegetables. This study involved a preliminary exploration of key biomarkers in the cold chain storage and transportation of these vegetables. This study aims to screen compounds with statistical differences and analyze their related metabolic pathways to elucidate the mechanisms by which metabolites affect vegetable freshness, thereby providing a theoretical basis for vegetable transportation and quality evaluation.

## 2. Materials and Methods

### 2.1. Sample Information

The experimental samples were provided by Shijiazhuang Huikang Food Co., Ltd. (Shijiazhuang, China). The sample number information is shown in Table 1. Four types of vegetable samples—chard, lettuce, crown daisy, and tomatoes—were selected for this study after harvesting. A total of 60 vegetables, all of similar shape and size, were stored in a refrigerator at 0 ± 1 °C with humidity controlled at 55% to 65% for durations of 0 days, 10 days, and 20 days, respectively. Every 10 vegetables were grouped together to create a representative sample. Prepare two samples for each storage condition. The vegetables were thoroughly washed and mixed, then homogenized using a blender to create a uniform sample. The homogenized material was stored in a −80 °C freezer for further analysis.

### 2.2. Reagents and Instruments

The reagents used included acetonitrile, methanol, n-butanol, acetyl chloride, methylamine double salt, pyridine, and BSTFA (all of chromatographic purity, purchased from CNW Technologies GmbH, Dusseldorf, Germany). Amino acid standards—arginine, ornithine, asparagine, alanine, glutamic acid, citrulline, arginine-N4, ornithine-D2, aspartate-D3, alanine-D4, glutamate-15N, and citrulline-D2—were obtained from Sigma and Dr. Ehrenstorfer, all with a purity of 95% or higher. The instruments used in the study were UPLC-Q Exactive Hybrid Quadrupole Orbitrap™ Mass Spectrometer (Thermo Fisher, Waltham, MA, USA); 5977B GC/MSD (Agilent, Santa Clara, CA, USA); WATERS ACQUITY UPLC-XEVO TQ-S Tandem Quadrupole Mass Spectrometer (WATERS, Milford, CT, USA); TDL-5-A centrifuge (Sigma, St. Louis, MO, USA); Eddy current oscillator (Scientific Industries, Tianjin, China); DK-8D electric constant temperature water bath (Shanghai Yiheng Technology Co., Ltd., Shanghai, China); vacuum drying oven (Beijing Suzi Technology, Beijing, China); Milli-Q Element Ultra-Pure Water System (Millipore Corporation, Boston, MA, USA); KQ-500E ultrasonic instrument; Electronic analytical balance (METTLER TOLEDO, Zurich, Switzerland); 0.22 μm filter membrane (Merck, Darmstadt, Germany).

### 2.3. Metabolite Extraction

#### 2.3.1. GC–MS Extraction Process

Accurately weigh 0.1 g of the sample into a 1.5 mL centrifuge tube. Add 1 mL of pre-cooled methanol at −20 °C and 20 µL of a 0.2 mg/mL ribitol internal standard. Vortex for 30 s, sonicate in an ice water bath for 30 min, and centrifuge at room temperature for 10 min (12,000 rpm). Transfer 500 µL of the supernatant to another clean centrifuge tube, then add 300 µL of chloroform and 500 µL of ultrapure water. Centrifuge at room temperature for 10 min (12,000 rpm), and vacuum dry 150 µL of the supernatant. Add 40 µL of methoxyamine hydrochloride pyridine solution (20 mg/mL) to the dried sample and incubate at 37 °C for 1 h in a constant temperature water bath. Then, add 50 µL of BSTFA and react for 40 min in a 50 °C water bath. After this, centrifuge for 10 min (12,000 rpm) and transfer the supernatant into an injection vial for analysis. Mix the supernatants of all samples in equal amounts to form a quality control (QC) sample.

#### 2.3.2. LC–MS/MS Extraction Process

Accurately weigh 0.1 g of the sample into a 1.5 mL centrifuge tube. Add 1 mL of pre-cooled methanol at −20 °C and 20 µL of a 0.2 mg/mL ribitol internal standard. Vortex for 30 s, sonicate in an ice water bath for 30 min, and centrifuge at room temperature for 10 min (12,000 rpm). Transfer 500 µL of the supernatant to another clean centrifuge tube, add 300 µL of chloroform and 500 µL of ultrapure water, and centrifuge at room temperature for 10 min (12,000 rpm). Take the supernatant and filter it through a 0.22 μm membrane for further analysis. Mix the supernatants of all samples in equal amounts to create a QC sample.

#### 2.3.3. Amino Acid Quantitative Detection and Extraction Process

Accurately weigh 0.1 g of the sample into a 1.5 mL centrifuge tube. Add 20 µL (10 µg/mL) of an amino acid internal standard mixture and 1 mL of acetonitrile solvent. Vortex for 30 s, sonicate for 30 min, and centrifuge at room temperature for 10 min (10,000 rpm). Transfer 300 µL of the supernatant to a nitrogen-blowing tube and dry under nitrogen. Add 100 µL of hydrochloric acid n-butanol derivatization reagent (n-butanol:acetyl chloride = 9:1, *v*/*v*), vortex for 2 min, and derivatize at 65 °C for 15 min. Blow dry with nitrogen, then add 300 µL of complex solution (water:acetonitrile = 8:2, *v*/*v*), filter through a 0.22 μm membrane, and prepare for machine detection.

### 2.4. Instrument Conditions

#### 2.4.1. GC–MS Analysis

The Agilent 5977B GC/MSD (Agilent, Santa Clara, CA, USA) was used with a DB-5ms capillary chromatography column (30 × 2.5 mm × 250 μm). The initial column temperature was set at 60 °C under programmed heating conditions and maintained for 2 min. The temperature was then increased to 180 °C at a rate of 15 °C/min, followed by an increase to 250 °C at a rate of 8 °C/min, and finally to 300 °C at a rate of 20 °C/min (held for 15 min). The injection port temperature was set at 250 °C. The carrier gas was high-purity helium, with an injection volume of 1 µL using a non-split injection method.

Mass spectrometry conditions included electron ionization (EI) mode, an ion source temperature of 250 °C, electron energy of 70 eV, solvent delay time of 5 min, and scanning mode set to full scan (*m*/*z* 30–500). QC samples were injected every three samples.

#### 2.4.2. LC–MS/MS Analysis

The UPLC-Q-Exactive liquid chromatography–mass spectrometer (Thermo Fisher, Waltham, MA, USA) was used with a C18 chromatographic column (length 10 cm, diameter 2.1 mm, particle size 1.7 μm). Mobile phase A consisted of a 0.1% formic acid aqueous solution, while mobile phase B was an acetonitrile solution. The linear UPLC gradient elution was as follows: 0 min at 2% B, 2 min at 2% B, 8 min at 50% B, 14 min at 98% B, 18 min at 98% B, 20.1 min at 2% B, and 25 min at 2% B. The flow rate was set at 0.3 mL/min, and the injection volume was 10 µL.

Mass spectrometry conditions included electrospray ionization (ESI) mode, an ion source temperature of 320 °C, collision energies (CE) of 10 V, 30 V, and 50 V, and full scan/data-dependent mass spectrometry scanning modes (Full MS/dd-MS2) for both positive and negative scans (*m*/*z* 70–1000). The target value for automatic gain control (AGC) was set to 3.0 × 106, with a spray voltage of 3.0 kV and a capillary temperature of 350 °C. QC samples were injected every three samples.

#### 2.4.3. Conditions for Amino Acid Quantitative Detection Instruments

The UPLC series quadrupole mass spectrometer was combined with an ADME chromatography column (150 mm × 2.1 mm, 5 μm) for analysis. (WATERS, Milford, USA) Mobile phase A was a 2.5 mM ammonium acetate solution with 0.1% formic acid, and mobile phase B was a 2.5 mM ammonium acetate solution with 0.1% formic acid in acetonitrile. The elution gradient was set as follows: 5% B at 0 min, 5% B at 1 min, 98% B at 6 min, 98% B at 7 min, 5% B at 7.01 min, and 5% B at 9 min. The flow rate was 0.3 mL/min, and the column temperature was maintained at 40 °C with an injection volume of 5 µL.

Mass spectrum conditions included an electrospray ion source, positive ion scanning, a capillary voltage of 0.5 kV, collision gas (argon) at a flow rate of 1.7 mL/min, and acquisition in segmented multi-reaction monitoring mode.

### 2.5. Statistical Analysis

The GC–MS raw data (.d) files were preprocessed using Unknowns Analysis software (Agilent, Santa Clara, CA, USA), including data file conversion (.cef), relative molecular weight (Mass), retention time (RT), and database search (NIST). Similarly, raw files extracted from the system were subjected to analysis and processing using MS-DIAL software (Developed jointly by Professor Masanori Arita’s team (RIKEN) and Professor Oliver Fiehn’s team (University of California, Davis)) [16]. This entailed peak extraction, alignment, filtering, and automatic integration. Create a multidimensional peak table containing information such as relative molecular weight (Mass), retention time (RT), peak area, etc. Two types of exported data were subjected to principal component analysis (PCA), orthogonal partial least squares discriminant analysis (OPLS-DA), and R^2^ and Q^2^ values were tested. Perform a *t*-test on the identified compounds using MPP (Mass Profiler Professional) software to obtain FC and *p* values. Take differential metabolites (*p* < 0.05, FC > 2 or FC < 0.5, VIP > 1) and map the differentially expressed metabolites to the Kyoto Encyclopedia of Genes and Genomes (KEGG) pathway database. Use MetaboAnalyst 6.0 [17] to comprehensively analyze pathways related to differentially expressed metabolites (including enrichment analysis and topological analysis) and locate key pathways highly correlated with metabolite differences.

## 3. Results

### 3.1. Principal Component Analysis (PCA)

This study conducted a non-targeted metabolomics analysis of the total metabolites in four types of vegetables under different storage conditions. Principal component analysis (PCA) was performed to reveal the overall metabolic differences and relationships among the samples. Using the UPLC-Q-Exactive liquid chromatography–mass spectrometer, peak area plots were generated for various metabolites.

The PCA results indicated significant differences in metabolite composition among the vegetable samples after refrigerated storage. The quality control (QC) group was positioned at the center of the graph, suggesting that the instrument maintained a stable state during the detection process. The contribution rates of the principal components were as follows: for the chard group, principal component 1 contributed 64.2%, and principal component 2 contributed 16.1%; for the lettuce group, principal component 1 contributed 57.3%, and principal component 2 contributed 16.6%; for the crown daisy group, principal component 1 contributed 58.5% and principal component 2 contributed 27%; and for the tomato group, principal component 1 contributed 59.7% and principal component 2 contributed 15%.

**Figure 1:**
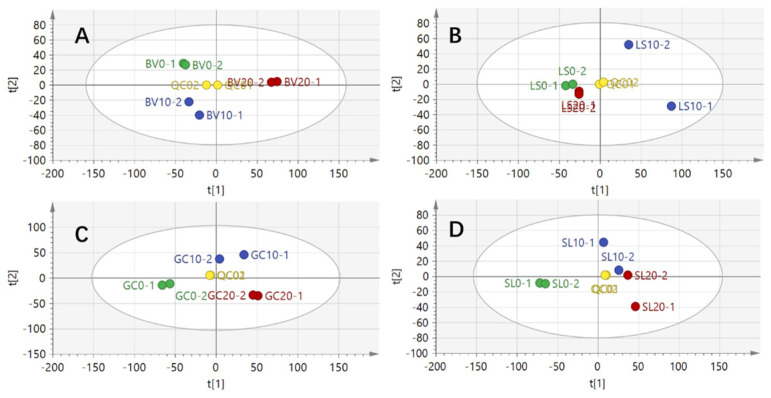
PCA diagram of cold chain storage for four types of vegetables: (**A**) chard, (**B**) lettuce, (**C**) crown daisy, (**D**) tomato.

These results demonstrate that the two principal components effectively capture the inter-group data information (Figure 1).

### 3.2. Partial Least Squares Discriminant Analysis (PLS-DA)

**Figure 2:**
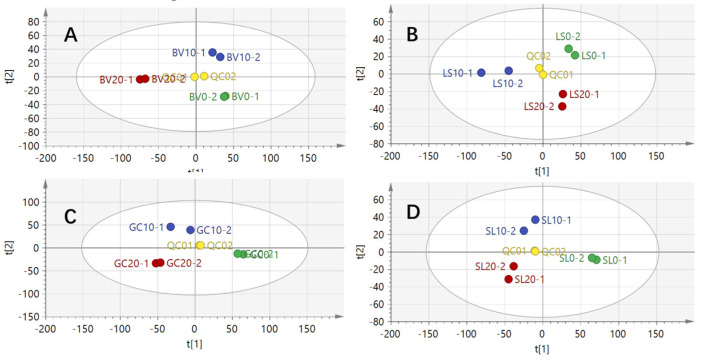
PLS-DA diagram of cold chain storage for four types of vegetables: (**A**) chard, (**B**) lettuce, (**C**) crown daisy, and (**D**) tomato.

While PCA is an unsupervised classification model, it can be influenced by inter-group and intra-group variations, resulting in a significant amount of original information being retained. To better highlight the differences between samples from different groups, the supervised classification model PLS-DA was used, and the results are shown in Figure 2. In this model, a greater horizontal distance between samples indicates a larger difference between groups, while a closer vertical distance suggests better repeatability within groups.

The explanatory variable R^2^ and the predictable variable Q^2^ for the chard group were 80.2% and 49.8%, respectively; for the lettuce group, they were 71.5% and 47.9%; for the crown daisy group, they were 85.5% and 46.3%; and for the tomato group, they were 74.8% and 42.6%. These values indicate strong interpretability and acceptable predictability across all models. The PLS-DA graph demonstrates significant differentiation among the samples, with all samples falling within the 95% confidence interval, confirming notable differences in the chemical composition of the vegetable samples after refrigeration.

**Figure 3:**
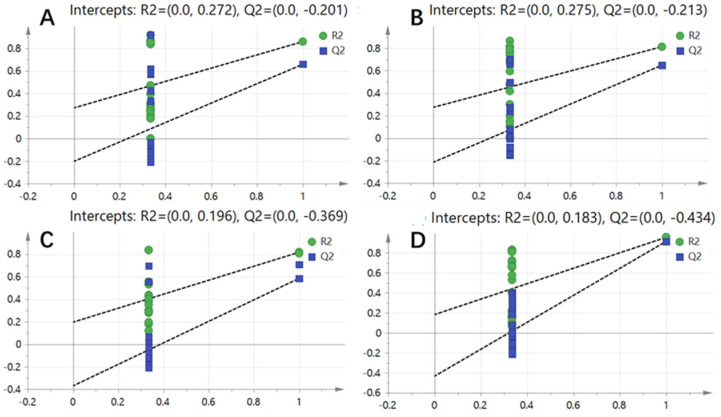
PLS-DA model permutation test results: (**A**) chard; (**B**) lettuce; (**C**) crown daisy; (**D**) tomato.

To further validate the reliability of the model, permutation testing was conducted, and the results are shown in Figure 3. The slopes of the R^2^ and Q^2^ regression lines were positive, and the intercept of the Q^2^ regression line was less than 0, indicating that the model is reliable and that there is no overfitting.

### 3.3. Screening of Differential Metabolites and Enrichment Analysis of Metabolic Pathways

**Figure 4:**
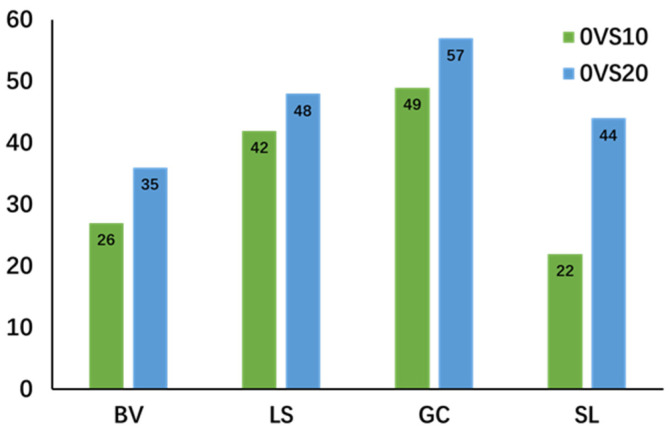
Quantity of Differential Metabolites (BV-chard; LS-lettuce; GC-crown daisy; SL-tomato).

In this study, vegetables stored for 0 days served as the control group, while those stored for longer periods were treated as experimental groups for pairwise comparison. To minimize false positive errors associated with using a single statistical analysis method, we combined variable importance (VIP > 1) from the PLS-DA model projections with fold changes (FC > 2 for upregulated and FC < 0.5 for downregulated) and *t*-test results (*p* < 0.05) to identify differential metabolites. The number of differential substances identified across the four comparison groups is presented in Figure 4, and detailed information on the differentially expressed substances can be found in the Supplementary Materials (Tables S1–S4).

The differential metabolites included a wide range of compounds such as amino acids, organic acids, fatty acids, sugars, flavonoids, and terpenoids. Changes in the content of these metabolites can significantly impact the taste and nutritional value of vegetables. To further explore the formation processes of different qualities and the primary pathways associated with these changes during storage, we mapped these differential substances to the KEGG pathway database. Key pathways identified that may influence vegetable metabolism include amino acid metabolism, the citric acid cycle, and carbohydrate metabolism.

Further topological analysis was conducted on these metabolic pathways, selecting key differential pathways with impact scores greater than 0.1 and *p* values < 0.05 (Table 2). The metabolic processes of various vegetables involve multiple pathways, with arginine biosynthesis and alanine, aspartate, and glutamate metabolism being notable common pathways. This indicates that amino acid metabolism plays a critical and universal role in the metabolic processes of vegetables.

### 3.4. Screening and Quantitative Detection of Key Differential Metabolites Affecting Metabolism

**Figure 5:**
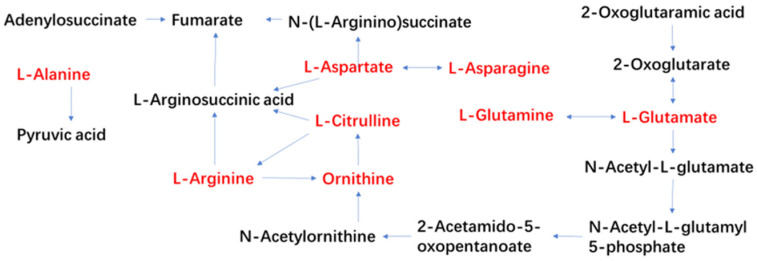
Metabolic pathway diagram of key differentially expressed substances.

Based on the pathway analysis results, we selected key metabolites for further investigation: 4 from chard, 5 from lettuce, 3 from crown daisy, and 5 from tomato. Specific information regarding these metabolites is summarized in Table 3. These metabolites primarily participate in two metabolic processes: arginine biosynthesis and the metabolism of alanine, aspartate, and glutamate. They may represent common key metabolites involved in the storage processes of these vegetables, as illustrated in Figure 5.

**Figure 6:**
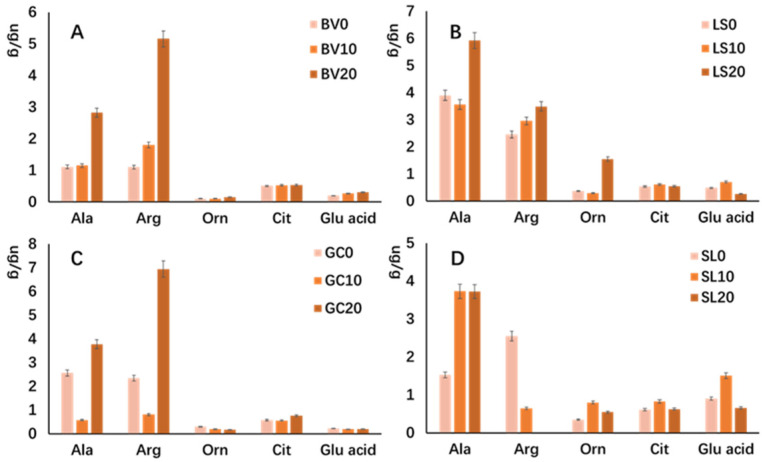
Detection results of key amino acid metabolites. (**A**) chard; (**B**) lettuce; (**C**) crown daisy; (**D**) tomato. (BV0 = Day 0; BV10 = Stored in the refrigerator for 10 days; BV20 = Stored in the refrigerator for 20 days; the other three are the same. Ala-alanine; Arg-arginine; Orn-ornithine; Cit-citrulline; Glu acid-glutamic acid).

To enhance the reliability of the experimental results, we conducted quantitative analyses on five amino acids to examine changes within these two metabolic pathways. The quantitative results are presented in Figure 6. To minimize ion suppression and enhance chromatographic separation and ionization efficiency, derivatization methods were employed for the detection of free amino acids. This method is based on the amino acid detection method proposed by Harder et al. and has been improved accordingly [18].

## 4. Discussion

Alanine is converted from pyruvic acid produced by the breakdown of carbohydrates, and further breakdown can generate malonic acid. Acetoacetate further decomposes alpha-ketoglutaric acid generated by the TCA cycle to produce glutamic acid. As shown in Figure 4, arginine synthesis involves a series of conversions, starting from glutamic acid to ornithine, citrulline, and finally arginine [19,20]. The biosynthesis rate of arginine is tightly regulated by various feedback mechanisms that depend on the overall nutritional status of the plant. Research indicates that the level of arginine in plants is controlled by multiple mechanisms, making it a vital amino acid for nitrogen storage. The catabolism of arginine not only mobilizes stored nitrogen but also contributes to the production of nitric oxide, polyamines, and potential proline [21,22].

Our experimental results demonstrated that the levels of alanine and arginine in chard, lettuce, and crown daisy showed varying degrees of increase during refrigeration, with consistent trends across these samples. This suggests that the nitrogen storage capacity of these vegetables is continuously enhancing, which may improve their stress resistance and tolerance. Additionally, this increase may be linked to enhanced signaling capabilities within the plants.

Glutamic acid is closely associated with primary nitrogen assimilation pathways in plants and acts as a signaling molecule regulating growth, development, and defense responses [23,24]. As a central molecule in amino acid metabolism in higher plants, glutamate can be converted into various amino acids, including arginine and proline, through enzymatic action [25,26]. Research has shown that glutamate can activate glutamate receptors (GLRs) in plant species, thereby regulating plant growth and development [27]. Glutamate also plays an important role in response to stress [28]. In our findings, the content of glutamic acid in lettuce and glutamine in tomatoes began to decrease after 10 days of refrigeration, indicating a gradual decline in their nitrogen storage capacity and signaling efficacy, which may lead to reduced stress resistance.

Citrulline is an important substance in the nitrogen metabolism pathway and stress resistance in vegetables and plays a role in maintaining normal crop growth under adverse stress [29,30,31]. Like arginine, nitrogen-rich citrulline is also an important part of endogenous nitrogen storage and transport in plants [32]. As shown in Figure 4, citrulline can be generated from glutamate or through the ornithine cycle. Citrulline can accumulate in large quantities under adverse conditions to maintain water status, eliminate excess free radicals, and protect green tissues from oxidative stress [29,33]. For example, Gong et al. found that genes involved in citrulline biosynthesis in tomatoes were significantly enriched under drought or salt stress [34].

From these observations, we conclude that by monitoring the levels of differential amino acids in vegetables—especially alanine, arginine, and glutamic acid—and comparing them with freshly picked vegetables, we can roughly infer the freshness, nutritional quality, and edibility of the produce. In the future, it is of great significance to explore preservation methods by spraying preservatives containing glutamic acid, arginine, etc. on fresh vegetables to maintain their freshness [35,36].

In addition to amino acids, other active ingredients, such as carbohydrates, also showed changes. For instance, we detected alterations in maltose content across all four vegetables, with an increase observed only in tomatoes while a slight decrease was noted in the other three. This is likely due to the ripening process of tomatoes after harvesting. Maltose is hydrolyzed from polysaccharides to produce glucose molecules, which can further influence the taste and flavor of vegetables [37]. Additionally, changes in the levels of glucose, fructose, and galactose affect vegetable taste. The test results indicated that these monosaccharides showed slight variations without significant increases or decreases. This study also detected the binding of flavonoids, such as quercetin and myricetin, with glycosides. These compounds have various beneficial effects, including anti-inflammatory and anti-cancer properties, making them highly advantageous for human health [38,39,40]. The results revealed a significant increase in the content of flavonoids in both chard and lettuce, indicating that these functional ingredients were retained after refrigeration. Many fatty acids, such as stearic acid, palmitic acid, and peanut acid, were also detected. Only a slight decrease in fatty acids was observed in the crown daisy, while no significant changes were noted in the other three vegetables. This suggests that refrigeration effectively inhibited fatty acid hydrolysis, thus maintaining the taste and quality of the vegetables.

In addition to the impact of storage temperature and time on the freshness and quality of vegetables, oxygen and carbon dioxide concentrations also play crucial roles. By controlling the environment to maintain high carbon dioxide levels and low oxygen concentrations, the respiration rate of vegetables can be reduced, minimizing polysaccharide consumption and inhibiting the synthesis and action of ethylene, which delays the ripening and aging processes. Low-oxygen environments can also suppress many microorganisms, helping to preserve the flavor, color, and nutritional value of vegetables [41,42]. Furthermore, 1-methylcyclopropene (1-MCP) is an effective ethylene inhibitor widely used to prevent the self-production of ethylene and premature ripening in ethylene-sensitive fruits and vegetables, thereby extending their freshness [43,44]. Potassium permanganate can oxidize ethylene, and porous materials treated with potassium permanganate solution can adsorb it [45]. Ozone gas also has the ability to oxidize ethylene and inhibit its biosynthesis [46]. Thus, by regulating oxygen and carbon dioxide levels in refrigeration units and incorporating ethylene-adsorbing devices, the freshness and nutritional value of vegetables can be effectively preserved.

In addition to the methods mentioned above, there are various techniques for preserving fruits and vegetables to extend their shelf life. For instance, coatings such as aloe gel or polysaccharide-based edible films can maintain the edible quality of different fruits and vegetables and extend their storage duration [47,48]. Additionally, technologies such as irradiation, ultraviolet (UV) irradiation, microwave treatment, and ultrasound have proven effective in delaying aging and maintaining quality [49,50]. Combining these technologies with methods for controlling environmental gases and temperatures can maximize their individual benefits and achieve more effective preservation.

## 5. Conclusions

This study utilized a combination of UHPLC-Q-Exactive and GC–MS techniques for qualitative and relative quantitative analysis of the metabolic products in vegetable samples, identifying significantly different substances across groups. The findings highlighted arginine biosynthesis and the metabolism of alanine, aspartate, and glutamate as key pathways in vegetable metabolism. Four key metabolites were selected from chard, five from lettuce, three from crown daisy, and five from tomato. Comparing the content of substances such as alanine and arginine can help infer the freshness and nutritional value of the vegetables, providing important references for detecting spoilage, determining storage time, and improving transportation conditions. Analyzing the content of these metabolites can provide insights into the freshness and nutritional value of vegetables, offering essential references for detecting deterioration, determining storage duration, and enhancing transportation conditions, thereby holding significant relevance for the vegetable transportation industry.

## Figures and Tables

**Table 1:** Sample Information.

	Storage Time/d	Sample Number
Chard	0	BV-0
	10	BV-10
	20	BV-20
Lettuce	0	LS-0
	10	LS-10
	20	LS-20
Crown daisy	0	GC-0
	10	GC-10
	20	GC-20
Tomato	0	SL-0
	10	SL-10
	20	SL-20

**Table 2:** Differential expression of metabolic pathways.

	Pathway Name	Total	Hits	*p*-Value	Impact
Chard	Arginine biosynthesis	18	4	0.00	0.22
	Alanine, aspartate and glutamate metabolism	22	4	0.00	0.18
	Indole alkaloid biosynthesis	4	2	0.00	0.50
Lettuce	Arginine biosynthesis	18	6	0.00	0.37
	Alanine, aspartate and glutamate metabolism	22	6	0.00	0.66
	Glyoxylate and dicarboxylate metabolism	29	5	0.00	0.22
	Arginine and proline metabolism	32	5	0.00	0.22
Crown daisy	Arginine biosynthesis	18	6	0.00	0.37
	Valine, leucine, and isoleucine biosynthesis	22	6	0.00	0.15
	Arginine and proline metabolism	32	6	0.00	0.24
	Alanine, aspartate and glutamate metabolism	22	5	0.00	0.54
	Galactose metabolism	27	5	0.00	0.14
	Citrate cycle (TCA cycle)	20	4	0.01	0.21
	Starch and sucrose metabolism	22	4	0.01	0.43
	C5-Branched dibasic acid metabolism	6	2	0.02	0.50
	Glyoxylate and dicarboxylate metabolism	29	4	0.02	0.16
	Cysteine and methionine metabolism	47	5	0.03	0.31
	Ascorbate and aldarate metabolism	20	3	0.04	0.16
	Carbon fixation in photosynthetic organisms	21	3	0.04	0.10
Tomato	Alanine, aspartate and glutamate metabolism	22	6	0.00	0.22
	Arginine biosynthesis	18	5	0.00	0.29
	Cysteine and methionine metabolism	47	7	0.00	0.44
	Pyruvate metabolism	23	5	0.00	0.29
	Pantothenate and CoA biosynthesis	25	5	0.00	0.16
	Sulfur metabolism	15	4	0.00	0.38
	Glyoxylate and dicarboxylate metabolism	29	5	0.00	0.18
	Citrate cycle (TCA cycle)	20	4	0.00	0.11
	Valine, leucine, and isoleucine biosynthesis	22	4	0.00	0.13
	Nitrogen metabolism	12	3	0.01	0.24
	Tyrosine metabolism	17	3	0.02	0.20
	Arginine and proline metabolism	32	4	0.02	0.11
	Glycine, serine and threonine metabolism	33	4	0.02	0.25
	Starch and sucrose metabolism	22	3	0.03	0.42

**Table 3:** Key Metabolites Involved in Pathways.

	Name	*p*-Value	FC	VIP	RT	MASS	Formula
Chard	L-Arginine	0.01	19.38	1.43	1.921	175.119	C6H14N4O2
	L-Ornithine	0.01	9.14	1.02	1.389	116.071	C5H12N2O2
	L-Asparagine	0.03	4.84	1.19	1.564	133.061	C4H8N2O3
	L-Alanine	0.03	−1.75	1.48	6.801	141.070	C3H7NO2
Lettuce	L-Ornithine	0.00	4.29	1.39	1.389	116.071	C5H12N2O2
	L-Glutamate	0.01	38.48	1.53	7.807	261.144	C11H20N2O5
	L-Asparagine	0.03	2.09	1.02	1.564	133.061	C4H8N2O3
	L-Arginine	0.02	9.19	1.18	1.921	175.119	C6H14N4O2
	L-Alanine	0.04	2.08	1.27	5.812	141.070	C3H7NO2
Crown daisy	L-Ornithine	0.00	2.85	1.24	1.389	116.071	C5H12N2O2
	L-Glutamate	0.00	0.09	1.30	7.807	261.144	C11H20N2O5
	L-Arginine	0.00	0.19	1.03	1.921	175.119	C6H14N4O2
Tomato	L-Asparagine	0.00	4.43	1.13	1.564	133.061	C4H8N2O3
	L-Alanine	0.00	3.40	1.27	5.812	43.13	C3H7NO2
	L-Glutamine	0.00	21.74	1.52	7.807	261.144	C11H20N2O5
	L-Citrulline	0.01	5.02	1.19	1.403	159.076	C6H13N3O3
	L-Ornithine	0.01	4.02	1.12	1.389	116.071	C5H12N2O2

## Data Availability

All of the data are contained within the article.

## Supplementary Materials

The following supporting information can be downloaded at: https://www.mdpi.com/article/10.3390/metabo14120665/s1, Table S1: Differentially expressed substances information of the chard group; Table S2: Differentially expressed substances information of the lettuce group; Table S3: Differentially expressed substances information of the crown daisy group; Table S4: Differentially expressed substances information of the tomato group.
