# Supplementary material for: Exploration of Freshness Identification Method for Refrigerated Vegetables Based on Metabolomics
Source: Metabolites. 2024 Dec 1;14(12):665. doi: 10.3390/metabo14120665 (PMC11676421; doi:10.3390/metabo14120665)
Supplement: Supplementary file 1 [file metabolites-14-00665-s001.zip › metabolites-3317215-supplementary.pdf]

Exploration of Freshness Identification Method for Refrigerated Vegetables Based on Metabolomics

Supplementary Materials

Table S1 Differentially expressed substances information of Genda group

	Compound	P Value	FC	RT	Mass	Formula
0vs10	Uridine	0.03	-1.61	1.44	243.06	C9 H12 N2 O6
	Pipecolic acid	0.01	-4.17	166.00	4.94	C13H15NO4
	Palmitoylcarnitine	0.00	-4.61	13.95	400.34	C23 H45 N O4
	Oleic acid	0.01	-3.54	20.48	281.25	C18 H34 O2
	Nonane	0.02	-10.58	57.00	7.58	C9H20
	Methylmalonic acid	0.02	-1.66	3.16	117.02	C4 H6 O4
	methadone	0.01	-2.26	20.76	316.95	C21 H18 [2]H9 N O
	l-Alanine	0.03	-1.75	141.07	6.80	C3 H7 N O2
	Isoquinoline	0.02	-1.77	9.78	130.07	C9 H7 N
	Guanidine	0.02	-4.35	87.06	5.16	C3H9N3
	Fexofenadine	0.03	-2.80	13.70	500.28	C32 H39 N O4
	Alanine	0.03	-1.75	141.07	6.80	C11H11F2NO3
	α,α-Trehalose	0.04	3.80	1.93	341.11	C12 H22 O11
	Safingol	0.00	2.54	12.60	302.31	C18 H39 N O2
	Quercetin	0.00	8.86	7.54	303.05	C15 H10 O7
	Oxoglutaric acid	0.02	1.69	2.65	145.01	C5 H6 O5
	Ornithine	0.01	3.81	1.19	131.08	C5 H12 N2 O2
	Norepinephrine	0.04	2.01	7.68	170.08	C8 H11 N O3
	Monobutyl phthalate	0.02	2.01	11.40	221.08	C12 H14 O4
	Methylumbelliferone	0.00	3.62	7.94	177.05	C10 H8 O3
	Methyl palmitate	0.02	4.19	11.53	288.29	C17 H34 O2
	Hydroxycaproic acid	0.01	2.98	8.14	131.07	C6 H12 O3
	Glucosamine	0.04	1.48	1.59	180.09	C6 H13 N O5
	D-Raffinose	0.03	5.59	1.37	527.16	C18 H32 O16
	DL-Tryptophan	0.00	2.61	7.47	205.10	C11 H12 N2 O2
	Carbonic acid	0.03	3.16	181.00	8.04	C13H13F5O3
0VS20	Glutathione	0.01	9.33	2.52	308.09	C10 H17 N3 O6 S
	Quinaldine	0.02	5.04	7.07	144.08	C10 H9 N
	Methylquinoline	0.01	16.14	7.83	144.08	C10 H9 N
	Indoleacrylic acid	0.01	8.00	7.34	188.07	C11 H9 N O2
	Isorhamnetin	0.01	16.91	8.60	317.07	C16 H12 O7
	theophylline	0.03	9.52	7.75	325.09	C16 H14 N4 O4
	acetic acid	0.04	8.03	4.07	332.13	C18 H21 N O3 S

Palmitoylcarnitine	0.03	-4.62	13.95	400.34	C23 H45 N O4
L-Serine	0.05	3.33	1.52	104.03	C3 H7 N O3
L-(-)-Serine	0.04	3.58	1.39	106.05	C3 H7 N O3
Dihydrouracil	0.02	4.36	1.37	156.08	C4 H6 N2 O2
3-Ureidopropionic acid	0.03	5.17	1.47	131.04	C4 H8 N2 O3
Asparagine	0.03	4.84	1.56	133.06	C4 H8 N2 O3
Butyl isothiocyanate	0.02	12.64	7.63	116.05	C5 H9 N S
Lysine	0.05	3.39	1.39	130.09	C6 H14 N2 O2
L-Arginine	0.01	19.38	1.92	175.12	C6 H14 N4 O2
L-Histidine	0.02	5.30	1.49	156.08	C6 H9 N3 O2
Isopropylmalic acid	0.02	24.77	7.89	175.06	C7 H12 O5
Indole	0.01	9.06	7.71	118.07	C8 H7 N
Uracil, 2TMS derivative	0.02	9.22	241.06	8.80	C10H20N2O2Si2
Undecane, 3,7-dimethyl-	0.02	6.53	43.00	9.18	C13H28
Terephthalic acid	0.03	2.64	317.20	20.74	C24H36O4
Phenylalanine, 2TMS derivative	0.01	11.65	73.15	11.39	C15H27NO2Si2
L-Asparagine	0.03	4.84	133.06	1.56	C4 H8 N2 O3
Octane, 3,5-dimethyl-	0.02	2.16	57.06	6.62	C10H22
L-Tryptophan, 3TMS derivative	0.01	5.52	202.00	17.34	C20H36N2O2Si3
2-Keto-l-gluconic acid, penta(O-trimethylsilyl)-	0.02	3.32	73.00	12.63	C21H50O7Si5
Fluvoxamine, N,N-bis(trimethylsilyl)-	0.01	6.16	174.22	10.31	C21H37F3N2O2Si2
2-Bromo dodecane	0.02	8.87	57.00	6.87	C12H26
Cuminyl alcohol, tert-butyldimethylsilyl ether	0.03	4.78	133.00	24.24	C16H28OSi
L-Ornithine	0.01	9.14	116.07	1.39	C5 H12 N2 O2
Nonane	0.02	-10.58	57.00	7.58	C9H20

Maltose	0.01	-4.89	73.16	14.85	C36H86O11Si8
L-Phenylalanine, 2TMS derivative	0.03	-3.79	73.16	11.39	C15H27NO2Si2
Guanidine, N,N-dimethyl-	0.02	-4.35	87.06	5.16	C3H9N3

Table S2 Differentially expressed substances information of lettuce group

	Compound	P Value	FC	RT	Mass	Formula
0vs10	Xanthine	0.05	2.08	1.45	153.04	C5 H4 N4 O2
	S-Adenosylmethionine	0.00	3.92	1.34	399.14	C15 H22 N6 O5 S
	Quercetin	0.04	15.58	7.54	303.05	C15 H10 O7
	Psychosine	0.04	2.51	8.19	462.35	C24 H47 N O7
	Phosphoric acid	0.03	4.43	1.88	98.98	H3 O4 P
	phenylpropiolic acid	0.03	2.03	7.77	147.04	C9 H6 O2
	Pantothenic acid	0.02	2.38	1.48	218.10	C9 H17 N O5
	Oleoyl ethanolamide	0.05	5.86	16.33	326.31	C20 H39 N O2
	Norepinephrine	0.00	10.85	7.68	170.08	C8 H11 N O3
	Naphthaleneacetamide	0.01	19.22	7.82	186.09	C12 H11 N O
	N6,N6,N6-Trimethyl-L-lysine	0.01	3.94	1.18	189.16	C9 H20 N2 O2
	N,N-dimethylarginine	0.01	2.26	1.36	203.15	C8 H18 N4 O2
	Myricetin 3-robinobioside	0.01	22.17	8.00	627.16	C27 H30 O17
	monomethyl phosphate	0.00	5.92	1.63	113.00	C H5 O4 P
	Methylimidazoleacetic acid	0.02	2.55	1.66	124.04	C6 H8 N2 O2
	Malonic acid	0.01	9.11	3.16	117.02	C4 H6 O4
	L-Tyrosine	0.01	3.47	3.49	182.08	C9 H11 N O3
	L-Threonic acid	0.00	3.61	1.44	135.03	C4 H8 O5
	L-Phenylalanine	0.05	3.58	1.46	166.09	C9 H11 N O2
	L-Isoleucine	0.00	4.34	2.82	132.10	C6 H13 N O2
	L-Glutamic acid	0.00	15.49	1.56	148.06	C5 H9 N O4
	L-gamma-Glutamyl-L-leucine	0.00	19.28	7.57	261.14	C11 H20 N2 O5
	Leucylproline	0.01	3.43	7.21	229.15	C11 H20 N2 O3
	L-cysteine	0.03	8.35	8.10	162.06	C6 H11 N O2 S
	L-Aspartic acid	0.00	3.80	1.56	134.04	C4 H7 N O4
	L-(+)-Tartaric acid	0.01	5.76	1.64	149.01	C4 H6 O6
	Kojic acid	0.01	2.72	1.47	143.03	C6 H6 O4
	Itaconic acid	0.00	27.42	3.02	111.01	C5 H6 O4
	Isocitric acid	0.01	7.05	1.84	191.02	C6 H8 O7
	Indoleacrylic acid	0.01	3.10	7.34	188.07	C11 H9 N O2
	Heptenophos	0.01	5.96	10.03	249.01	C9 H12 Cl O4 P
	glutamine	0.01	38.48	1.51	145.06	C5 H10 N2 O3

	gamma-Acetylenic gaba	0.01	2.71	2.04	128.07	C6 H9 N O2
	Galacturonic acid	0.00	-4.91	1.56	193.03	C6 H10 O7
	Epinephrine	0.01	-4.12	7.19	184.10	C9 H13 N O3
	D-Saccharic acid	0.02	-4.22	1.55	209.03	C6 H10 O8
	L-Arginine	0.02	9.19	1.92	175.12	C6 H14 N4 O2
	Aspartic acid, 3TMS derivative	0.01	3.86	10.39	232.00	C13H31NO4Si3
	Asparagine, 4TMS derivative	0.01	1.85	11.36	73.00	C16H40N2O3Si4
	Argininosuccinic acid	0.04	-5.04	1.82	291.13	C10 H18 N4 O6
	Arachidic acid, TMS derivative	0.01	-7.02	19.56	117.05	C23H48O2Si
	Aconitic acid	0.00	40.39	3.03	175.02	C6 H6 O6
	Vigabatrin	0.03	3.95	1.10	130.09	C6 H11 N O2
	Val-Leu	0.00	-3.16	7.29	231.17	C11 H22 N2 O3
	Tryptamine	0.02	13.68	7.72	144.08	C10 H12 N2
	Tilarginine	0.04	2.28	1.48	189.13	C7 H16 N4 O2
	Phosphocholine	0.03	3.21	13.53	184.07	C5 H14 N O4 P
	NN-Dimethylthioacetamide	0.01	-2.71	7.25	104.05	C4 H9 N S
	N6,N6,N6-Trimethyl-L-lysine	0.01	4.59	1.18	189.16	C9 H20 N2 O2
	N,N-dimethylarginine	0.01	3.13	1.36	203.15	C8 H18 N4 O2
	N-(tert-Butylcarbamoyl)-3-methyl-L-valine	0.03	-2.34	7.56	231.17	C11 H22 N2 O3
	Myricetin 3-robinobioside	0.03	3.07	8.00	627.16	C27 H30 O17
0vs20	L-Glutamate	0.01	38.48	7.81	261.14	C11 H20 N2 O5
	monomethyl phosphate	0.01	2.11	1.63	113.00	C H5 O4 P
	Methyl palmitate	0.00	30.17	11.53	288.29	C17 H34 O2
	Meglutol	0.00	4.29	1.53	145.05	C6 H10 O5
	Maltotetraose	0.01	-4.47	1.37	689.21	C24 H42 O21
	L-Asparagine	0.03	2.09	1.56	133.06	C4 H8 N2 O3
	Maleamic acid	0.02	2.18	1.55	116.03	C4 H5 N O3
	L-Tyrosine	0.00	3.66	3.49	182.08	C9 H11 N O3
	L-Isoleucine	0.00	3.17	2.82	132.10	C6 H13 N O2
	Linoleoyl ethanolamide	0.01	2.24	15.47	324.29	C20 H37 N O2
	L-Alanine	0.04	2.08	5.81	141.07	C3H7NO2
	L-Glutamic acid	0.00	4.28	1.56	148.06	C5 H9 N O4
	L-gamma-Glutamyl-L-leucine	0.02	2.66	7.57	261.14	C11 H20 N2 O5
	L-Aspartic acid	0.01	2.06	1.56	134.04	C4 H7 N O4

L-arginine	0.05	2.25	1.92	175.12	C6 H14 N4 O2
L-(+)-Tartaric acid	0.04	3.39	1.64	149.01	C4 H6 O6
Itaconic acid	0.01	2.47	3.02	111.01	C5 H6 O4
Guanine	0.02	-3.32	1.37	152.06	C5 H5 N5 O
glutamine	0.02	20.55	1.33	145.06	C5 H10 N2 O3
Gluconic acid	0.00	-3.07	1.63	195.05	C6 H12 O7
D-Raffinose	0.01	-5.53	1.37	527.16	C18 H32 O16
DL-Tryptophan	0.01	2.37	7.47	205.10	C11 H12 N2 O2
L-Ornithine	0.00	4.29	1.39	116.07	C5 H12 N2 O2
DL-Malic acid	0.01	2.20	1.71	133.01	C4 H6 O5
D-(+)-Proline	0.00	6.47	1.40	116.07	C5 H9 N O2
D-(+)-Maltose	0.01	-2.60	1.52	365.11	C12 H22 O11
D-(+)-Malic acid	0.01	2.20	1.48	133.01	C4 H6 O5
Cytosine	0.00	-4.06	1.40	112.05	C4 H5 N3 O
Cys-pro	0.00	4.23	8.40	219.08	C8 H14 N2 O3 S
Citric acid	0.01	2.87	1.84	191.02	C6 H8 O7
Celecoxib	0.02	8.13	13.32	382.08	C17 H14 F3 N3 O2 S
Butyl isothiocyanate	0.04	4.07	7.63	116.05	C5 H9 N S
Betazole	0.02	4.25	1.34	112.09	C5 H9 N3
Aspartame	0.00	4.10	8.37	295.13	C14 H18 N2 O5
Andrographolide	0.02	-3.27	12.48	333.20	C20 H30 O5
3-Ureidopropionic acid	0.05	2.06	1.47	131.04	C4 H8 N2 O3
3-(tetradecyloxy)propylamine	0.02	16.17	12.88	272.29	C17 H37 N O
2-Isopropylmalic acid	0.00	3.76	7.89	175.06	C7 H12 O5

Table S3 Differentially expressed substances information of Tonghao group

	Compound	P Value	FC	RT	Mass	Formula
0vs10	α-Eleostearic acid	0.03	-0.42	11.96	279.23	C18 H30 O2
	Valylproline	0.04	-0.38	1.46	215.14	C10 H18 N2 O3
	Valine	0.01	-0.76	1.82	118.09	C5 H11 N O2
	Tryptamine	0.01	-0.12	7.72	144.08	C10 H12 N2
	Tilarginine	0.00	-0.20	1.48	189.13	C7 H16 N4 O2
	Threonine	0.03	-2.98	1.29	118.05	C4 H9 N O3
	Terephthalic acid	0.01	-0.31	8.15	165.02	C8 H6 O4
	Stearoyl Ethanolamide	0.01	-0.27	13.87	310.31	C20 H41 N O2
	Stearic Acid	0.00	-0.41	13.30	302.31	C18 H36 O2
	Stearic acid	0.00	-0.44	13.13	302.30	C18 H36 O2
	S-Adenosylmethionine	0.04	-0.74	1.34	399.14	C15 H22 N6 O5 S
	Quercetin	0.01	-0.38	8.75	303.05	C15 H10 O7
	Pyruvic acid	0.01	-0.36	2.12	87.01	C3 H4 O3

	Prolylleucine	0.01	-0.42	1.65	229.15	C11 H20 N2 O3
	Proline	0.05	-0.45	7.53	116.07	C5 H9 N O2
	peroxyoxalate	0.03	-0.32	7.33	104.98	C2 O5
	Palmitoleic Acid	0.01	-0.34	14.76	277.22	C16 H30 O2
	Palmitic diethanolamide	0.00	-0.12	12.96	344.32	C20 H41 N O3
	Palmitic Acid	0.02	-0.24	14.42	274.27	C16 H32 O2
	Ornithine	0.02	-0.44	1.19	131.08	C5 H12 N2 O2
	O-Methylpongamol	0.04	-0.83	2.75	309.11	C19 H16 O4
	Oleoyl ethanolamide	0.00	-0.32	11.95	332.32	C20 H39 N O2
	Nicotinic acid	0.01	-0.60	1.60	124.04	C6 H5 N O2
	N-Ethylglycine	0.00	-0.25	1.37	102.05	C4 H9 N O2
	N-Caprylyldiethanolamine	0.03	-0.49	9.25	218.21	C12 H27 N O2
	N-Benzylformamide	0.00	-0.18	3.01	136.08	C8 H9 N O
	Naphthaleneacetamide	0.02	-0.60	7.43	186.09	C12 H11 N O
	N6,N6,N6-Trimethyl-L-lysine	0.00	-0.25	1.18	189.16	C9 H20 N2 O2
	Methylmalonic acid	0.01	-4.93	3.16	117.02	C4 H6 O4
	Methylimidazoleacetic acid	0.01	-0.54	1.66	124.04	C6 H8 N2 O2
	Methyl palmitate	0.00	-0.45	11.78	288.29	C17 H34 O2
	L-Tyrosine	0.00	-0.02	3.01	182.08	C9 H11 N O3
	L-Proline	0.02	-0.61	6.95	116.07	C5 H9 N O2
	L-Phenylalanine	0.00	-0.32	6.96	166.09	C9 H11 N O2
	L-Methionine	0.00	-0.04	1.45	150.06	C5 H11 N O2 S
	L-Isoleucine	0.00	-0.10	1.45	132.10	C6 H13 N O2
	Linoleamide	0.00	-0.15	12.97	280.26	C18 H33 N O
	L-Histidine	0.01	-0.07	1.95	156.08	C6 H9 N3 O2
	L-Glutamic acid	0.00	-0.28	1.39	148.06	C5 H9 N O4
	Leucylproline	0.03	-0.16	7.54	229.15	C11 H20 N2 O3
	L-Aspartic acid	0.01	-9.63	1.40	132.03	C4 H7 N O4
	L-(+)-Citrulline	0.02	-0.16	1.32	198.08	C6 H13 N3 O3
	L-(-)-Serine	0.04	-0.80	1.52	106.05	C3 H7 N O3
	L(-)-Pipecolinic acid	0.00	-0.22	1.43	130.09	C6 H11 N O2
	L-(-)-Methionine	0.04	-0.27	7.22	150.06	C5 H11 N O2 S
	Guanine	0.02	-3.92	1.73	152.06	C5 H5 N5 O
	L-Arginine	0.00	0.19	1.92	175.12	C6 H14 N4 O2
	L-Rhamnose, 4TMS derivative	0.00	7.01	14.23	204.00	C18H44O5Si4
	Lactose, 8TMS derivative	0.01	-3.21	21.42	361.00	C36H86O11Si8
0vs20	Homo-L-arginine	0.00	-0.19	1.30	189.13	C7 H16 N4 O2
	Pipecolinic acid	0.00	-0.48	2.11	130.09	C6 H11 N O2

Malic acid	0.00	-2.39	1.71	133.01	C4 H6 O5
Stearic acid	0.00	-0.44	13.13	302.30	C18 H36 O2
Arginine	0.00	-0.09	1.92	175.12	C6 H14 N4 O2
Lipoic acid	0.00	-0.16	1.44	205.03	C8 H14 O2 S2
Arachidic acid	0.00	4.68	19.56	369.16	C23H48O2Si
D-Glucose	0.00	-0.26	1.59	261.04	C6 H13 O9 P
Gluconic acid	0.00	-0.28	1.63	195.05	C6 H12 O7
Malic acid	0.00	-2.68	1.99	133.01	C4 H6 O5
Quercetin 3-glucoside 7-xyloside	0.00	-0.25	8.68	463.09	C21 H20 O12
Leucylproline	0.00	-0.30	6.95	229.15	C11 H20 N2 O3
Prolylglycine	0.00	-0.18	7.93	171.08	C7 H12 N2 O3
Psychosine	0.00	0.00	8.86	462.35	C24 H47 N O7
Indoleacrylic acid	0.00	0.28	7.34	188.07	C11 H9 N O2
Phenylalanine	0.00	-0.35	7.81	266.14	C14 H19 N O4
Dodecane	0.00	2.83	8.39	71.00	C15H32
Quercetin	0.00	-0.12	7.72	303.05	C15 H10 O7
Maltotetraose	0.00	-0.06	1.37	689.21	C24 H42 O21
Phenylalanine	0.00	-0.07	7.65	164.07	C9 H11 N O2
Heptenophos	0.00	-0.14	10.03	249.01	C9 H12 Cl O4 P
Methionine	0.00	-0.02	1.45	150.06	C5 H11 N O2 S
Histidine	0.00	-0.02	1.95	156.08	C6 H9 N3 O2
Adenosine	0.00	0.00	4.65	268.10	C10 H13 N5 O4
Linoleamide	0.00	-0.29	12.85	280.26	C18 H33 N O
L-Proline	0.00	-2.01	1.53	116.07	C5 H9 N O2
Sulfasalazine	0.00	-0.11	10.13	437.04	C18 H14 N4 O5 S
Methionine	0.00	-0.15	7.09	150.06	C5 H11 N O2 S
Propionylcarnitine	0.00	-0.09	3.87	218.14	C10 H19 N O4
Octadecane	0.00	1.17	18.80	71.07	C18H37I
Eleostearic acid	0.00	0.32	14.34	279.23	C18 H30 O2
Oleoyl ethanolamide	0.00	-0.32	11.95	332.32	C20 H39 N O2
Phosphocholine	0.00	-0.08	1.37	184.07	C5 H14 N O4 P
Lactic Acid	0.00	-0.40	1.34	89.02	C3 H6 O3
Maltose	0.00	-0.06	1.69	365.11	C12 H22 O11
L-Glutamate	0.00	0.09	7.81	261.14	C11 H20 N2 O5
Furoic acid	0.00	-0.16	2.94	111.01	C5 H4 O3
Citric acid	0.00	-0.17	3.09	191.02	C6 H8 O7
Methohexital	0.00	-0.16	7.82	263.14	C14 H18 N2 O3
Oxoglutaric acid	0.00	-0.20	2.65	145.01	C5 H6 O5
Lactose	0.00	3.21	21.42	361.00	C36H86O11Si8
N6,N6,N6-Trimethyl-L-lysine	0.00	-0.25	1.18	189.16	C9 H20 N2 O2
Xanthurenic acid	0.00	0.04	1.33	206.05	C10 H7 N O4

Taurine	0.00	-0.03	1.36	124.01	C2 H7 N O3 S
Pipecolinic acid	0.00	-0.25	1.43	130.09	C6 H11 N O2
Betaine	0.00	-0.19	1.38	118.09	C5 H11 N O2
Isopropylmalic acid	0.00	-0.03	7.89	175.06	C7 H12 O5
Glutamic acid	0.00	-0.09	1.62	146.04	C5 H9 N O4
aspartic acid	0.00	-0.11	1.55	176.06	C6 H9 N O5
Hypoxanthine	0.00	-0.03	1.89	137.05	C5 H4 N4 O
Methylxanthine	0.00	-0.05	1.43	165.04	C6 H6 N4 O2
L-Ornithine	0.00	2.85	1.39	116.07	C5 H12 N2 O2
Lysine	0.00	-0.22	1.39	130.09	C6 H14 N2 O2
L-Aspartic acid	0.00	-0.11	1.64	132.03	C4 H7 N O4
Valine	0.00	0.22	1.67	118.09	C5 H11 N O2
L-leucine	0.00	-0.02	6.92	232.15	C11 H21 N O4
Arabinofuranose	0.00	6.03	19.86	217.00	C17H42O5Si4

Table S4 Differentially expressed substances information of tomato group

	Compound	p	FC	RT	Mass	Formula
0vs10	N-P-Tosylglycine	0.00	17.69	6.87	230.05	C9 H11 N O4 S
	N-Benzylformamide	0.00	37.80	3.01	136.08	C8 H9 N O
	N6,N6,N6-Trimethyl-L-lysine	0.00	2.53	1.18	189.16	C9 H20 N2 O2
	N-(Butoxyacetyl)glutamine	0.00	211.74	7.81	261.14	C11 H20 N2 O5
	Maltotetraose	0.00	7.16	1.37	689.21	C24 H42 O21
	Luteolin 7-lactate	0.00	2.24	10.98	357.06	C18 H14 O8
	Leucylproline	0.00	4.96	7.21	229.15	C11 H20 N2 O3
	L(-)-Pipecolinic acid	0.00	2.73	1.55	130.09	C6 H11 N O2
	L-(-)-Methionine	0.00	6.38	7.09	150.06	C5 H11 N O2 S
	DL-Lactic Acid	0.00	3.01	1.34	89.02	C3 H6 O3
	Demethylwedelolactone	0.00	81.52	9.76	299.02	C15 H8 O7
	Boc-L-Phenylalanine	0.00	5.96	7.81	266.14	C14 H19 N O4
	Adenosine	0.00	25.29	4.90	268.10	C10 H13 N5 O4
	Adenine	0.00	1.91	4.63	136.06	C5 H5 N5
	6-Methoxyquinoline	0.00	51.64	5.68	160.08	C10 H9 N O
	4-Methoxyphenol, TMS derivative	0.00	-6.60	6.28	196.12	C10H16O2Si
	.beta.-D-Mannopyranuronic acid, 5TMS	0.00	-5.25	21.85	204.00	C21H50O7Si5
	Boric acid, 3TMS derivative	0.00	-1.61	5.47	221.00	C9H27BO3Si3
	Hexadecane	0.00	-1.53	10.27	57.07	C16H34
	d-Fructose, 1,3,4,5-tetrakis-O-(trimethylsilyl)-, o-methyloxime, 6-[bis(trimethylsilyl) phosphate]	0.00	-7.13	18.25	315.00	C25H64NO9PSi6
	.alpha.-D-Glucopyranuronic acid, 5TMS derivative	0.01	-1.55	22.10	204.00	C21H50O7Si5
	D-Allose, oxime (isomer 2), 6TMS derivative	0.01	6.42	16.54	73.00	C24H61NO6Si6
0vs20	L-Alanine	0.00	3.29	5.81	43.13	C7H14N2O2
	L-Glutamine	0.00	21.74	7.81	261.14	C11 H20 N2 O5
	L-gamma-Glutamyl-L-leucine	0.01	49.56	7.57	261.14	C11 H20 N2 O5
	propanoic acid	0.00	36.41	3.08	165.05	C9 H10 O4

Guanine	0.00	31.15	1.37	152.06	C5 H5 N5 O
Methacrolein	0.01	18.72	10.44	145.01	C3 H4 S
Argininosuccinic acid	0.00	18.28	1.82	291.13	C10 H18 N4 O6
Leucylproline	0.00	15.19	7.21	229.15	C11 H20 N2 O3
Homocysteine	0.00	13.67	3.99	119.02	C4 H9 N O2 S
Methionine	0.00	13.31	7.09	150.06	C5 H11 N O2 S
Butyl isothiocyanate	0.01	13.24	7.63	116.05	C5 H9 N S
cysteine	0.01	12.45	8.10	162.06	C6 H11 N O2 S
Valylproline	0.00	12.38	7.38	215.14	C10 H18 N2 O3
Thiomorpholine 3-carboxylate	0.01	10.79	1.55	104.05	C4 H9 N S
ethanol	0.00	10.48	9.60	129.04	C6 H8 O S
Tryptophan	0.00	9.31	7.47	205.10	C11 H12 N2 O2
propanoic acid	0.01	8.96	7.63	343.10	C15 H20 O9
Tyrosine	0.00	8.70	3.49	182.08	C9 H11 N O3
butenoic acid	0.00	8.65	1.40	191.10	C7 H14 N2 O4
Indoleacrylic acid	0.00	7.55	7.34	188.07	C11 H9 N O2
L-valine	0.00	7.39	7.56	231.17	C11 H22 N2 O3
Maltotetraose	0.00	7.16	1.37	689.21	C24 H42 O21
Adenosylmethionine	0.01	6.48	1.34	399.14	C15 H22 N6 O5 S
Malic acid	0.00	6.41	1.48	133.01	C4 H6 O5
Maltose	0.00	6.22	1.52	365.11	C12 H22 O11
L-Phenylalanine	0.00	5.96	7.81	266.14	C14 H19 N O4
Isoleucine	0.00	5.55	2.82	132.10	C6 H13 N O2
Sinapinic acid	0.00	5.46	14.39	113.04	C11 H12 O5
L-Citrulline	0.01	5.02	1.40	159.08	C6 H13 N3 O3
Pipecolic acid	0.01	5.02	1.42	159.08	C6 H10 N2 O3
Proline	0.00	4.75	7.53	116.07	C5 H9 N O2
Pyroglutamic Acid	0.00	4.63	7.75	130.05	C5 H7 N O3
Ciprofloxacin	0.01	4.55	3.09	332.13	C17 H18 F N3 O3
N,N-dimethylarginine	0.00	4.54	1.36	203.15	C8 H18 N4 O2
Isocitric acid	0.01	4.48	1.84	191.02	C6 H8 O7
L-Asparagine	0.00	4.43	1.56	133.06	C4 H8 N2 O3
Pantothenic acid	0.01	4.40	1.48	218.10	C9 H17 N O5
Serine	0.00	4.17	1.39	106.05	C3 H7 N O3
Pyruvic acid	0.00	4.17	1.71	87.01	C3 H4 O3
trimethadione	0.00	4.12	1.34	144.07	C6 H9 N O3
Mesalamine	0.01	4.10	3.98	154.05	C7 H7 N O3
Methylxanthine	0.01	4.09	1.43	165.04	C6 H6 N4 O2
L-Ornithine	0.01	4.02	1.39	116.07	C5 H12 N2 O2
Proline	0.01	4.02	1.40	116.07	C5 H9 N O2
